# Preparation of nanodiamond anchored on copper tannic acid as a heterogenous catalyst for synthesis of 1,4-benzodiazepines derivatives

**DOI:** 10.1038/s41598-024-58563-0

**Published:** 2024-04-15

**Authors:** Reza Ghalavand, Hossein Ghafuri, Hadi Hassani Ardeshiri

**Affiliations:** Catalysts and Organic Synthesis Research Laboratory, Department of Chemistry, Iran University of Science and Technology, Tehran, 16846-13114 Iran

**Keywords:** Nanodiamond, Benzodiazepine, Tannic acid, Heterogeneous catalyst, Esterification process, Catalyst synthesis, Catalytic mechanisms, Heterogeneous catalysis

## Abstract

In this research, a new and eco-friendly heterogeneous catalyst (ND@Tannicacid-Cu) was synthesized based on nanodiamond and copper tannic acid via esterification process. The as-prepared catalyst was characterized by Fourier transforms infrared spectroscopy (FT-IR), energy dispersive X-ray spectroscopy (EDX), scanning electron microscopy (SEM), and X-ray diffraction (XRD) methods. The catalytic efficacy of the intended catalyst was examined by one-step three-component reaction of 1,4-benzodiazepine derivatives from a mixture of ortho-phenylenediamine, aromatic aldehydes, and dimedone under mild conditions. In all instances, corresponding 2,4-benzodiazepines derivatives were synthesized with high efficiency, short reaction time, straightforward work up procedure, no requirement for column-chromatography, and cost-effective catalyst. The heterogeneous catalyst was easily recycled using fillers, and it can be reused for eight cycles without significantly diminishing its performance.

## Introduction

Different forms of carbon exist, with nanodiamonds, graphene, carbonitrile, fullerene carbon nanotubes being the most significant among them. These diverse forms of carbon find application in various fields^1,2^. Recently, there has been an increasing emphasis on non-molecular carbon structures, particularly detonation nanodiamonds (NDs)^3,4^. The unique characteristic that distinguishes detonation nanodiamonds from fullerenes and carbon nanotubes is their utilization as a base for metal clusters^5^. Unlike fullerenes and carbon nanotubes, detonation nanodiamonds lack double bonds whose base orbitals could interact with the supported metals^6^. The NDs nanomaterials possess a unique and distinct structural configuration that distinguishes them in the realm of advanced materials. At their nucleus, NDs demonstrate SP^3^ hybridization, a distinct arrangement of carbon atoms that imparts exceptional stability and resilience^7^. This core structure is further enveloped by multiple layers of SP^2^ carbons, creating a highly intriguing composite. The defining feature of nanodiamonds, and one that profoundly influences their versatility, lies in the presence of these SP^2^ carbons on their surface. This characteristic is pivotal and underpins the unique properties of nanodiamonds. This unique characteristic makes NDs highly suitable for the production of hybrid materials. The remarkable surface properties of NDs have broad-reaching implications, rendering them highly adaptable and valuable across a myriad of fields^8^. Based on the aforementioned instances, the utilization of diamond nanoparticles as supports for metals holds significant significance in the development of organo-metallic catalysts and the exploration of their catalytic performance in a range of reactions^9^. The metals that are affixed to nanodiamond, e.g., copper, nickel, iron, palladium, and rhodium have demonstrated efficacy in various reactions, including oxidation processes, hydrogenation of carbon–carbon double-triple bonds, and hydrochlorination reactions^10,11^. Tannic acid, a plant polyphenol, is readily present in all aerial plant tissues. In the past, tannic acid was utilized to address diarrhoea and skin burns, and also administered rectally to treat unspecified rectal disorders^12–14^. The challenge of extracting tannic acid from a reaction after it has been used is overcome by its interaction with heterogeneous nanoparticles^15–22^. This interaction aims to address the issue concerning the water solubility of tannic acid, which would otherwise present a difficulty in the process of separating it from the reaction mixture. Multicomponent reactions (MCRs) can be regarded as a highly effective means to attain this objective, as these reactions possess key attributes, such as the employment of eco-friendly solvents, reduced energy consumption, optimal utilization of atoms, expedited reaction times, and excellent efficacy^23^. Benzodiazepines are a category of sedative substances characterized by their molecular composition consisting of the amalgamation of a benzene ring and a diazepine ring. These compounds serve as essential intermediates for the production of heterocyclic compounds, such as oxadiazole^24^, triazole^25^, oxazine^26^, and furanodiazepines^27^. Furthermore, benzodiazepine derivatives have found wide-ranging use due to their therapeutic properties, such as anticonvulsants^28^, sedatives^29^, and antidepressants^30^. Various catalysts have been reported for benzodiazepine synthesis, such as CoFe_2_O_4_@GO–K22-Ni nanocomposite^31^, graphene oxide (GO) nanosheets^31^, palladium (Pd)-catalyzed approaches^32^, Fe_3_O_4_/f-MWCNT/Ni_2_B^33^, and etc. While these methods have demonstrated promise in benzodiazepine synthesis, it is imperative to acknowledge their associated limitations. Common issues that have been encountered include prolonged reaction times, low yields, challenges in catalyst separation, harsh reaction conditions, and high energy consumption. Recognizing these limitations, this study aims to address these shortcomings and propose an innovative approach that overcomes these challenges while ensuring efficient and sustainable benzodiazepine synthesis. Herein, we introduce a new and efficient heterogeneous catalyst, denoted as Nanodiamond@Tannic acid (ND@ tannic acid), which is functionalized with Cu nanoparticles for the first time^34,35^. The ND@Tannicacid-Cu catalyst was employed in the synthesis of 1,4-benzodiazepine derivatives. It can be easily separated from the reaction mixture through filtration and demonstrates the ability to be reused for at least eight cycles without experiencing a significant decrease in catalytic activity. Importantly, this nanocatalyst possesses intrinsic properties, such as eco-friendly, biocompatibility, recyclability, physiological inertness, and non-toxicity.

## Experimental

### Materials and methods

All of the solvents, chemicals, and reagents were procured from Merck, Sigma, and Aldrich. The spherical detonation NDs were obtained from plasma chem company (Germany). Fourier transform infrared spectroscopy (FT-IR) was recorded on a Shimadzu IR-470 spectrometer by the KBr pellet. Melting points were measured on an Electrothermal 9100 device. 1H nuclear magnetic resonance (NMR) spectra were recorded on a Bruker DRX-500 Avance spectrometer at 500 ( Figures. S1–S5). SEM images were acquired using a Sigma-Zeiss microscope equipped with an attached camera. The nanocatalyst underwent elemental analysis through the utilization of energy-dispersive X-ray (EDX) analysis, which was recorded via the Numerix DXP-X10P.

### Preparation of ND@Tannicacid-Cu catalyst

**Figure 1:**
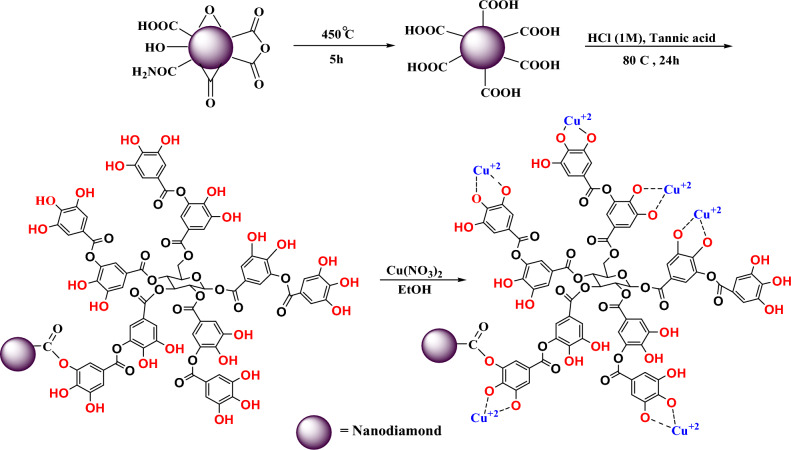
Immobilizing of copper (Cu) onto tannic acid. The process of immobilizing copper (Cu) onto tannic acid occurs in two distinct steps. Firstly, diamond nanoparticles are carboxylated through a heat-induced reaction, followed by the esterification of these functionalized carboxylated nanoparticles with tannic acid. In the final step, copper (Cu) is securely anchored to the tannic acid. ND@Tannicacid-Cu proves to be highly effective for the synthesis of 1,4-benzodiazepine derivatives.

In order to synthesize carboxylate nanodiamonds (ND), 1.00 g of diamond nanoparticles (NPs) was placed in the oven at a temperature of 450 °C for 5 h at a rate of 1 °C/min. In the process of esterification of nanodiamond functionalized with tannic acid (Fig. 1), 10 mg of nanodiamond functionalized with carboxylic acid was mixed in 10 mL of deionized water and then stirred at 80 °C for 5 h. Afterward, HCl (1 M) was added to the reaction until the pH value reached 4.7. After that, 20 mg of tannic acid was added drop-wise to the above reaction mixture. The resultant mixture was stirred for 24 h at 80 °C. Finally, the prepared nanoparticle was filtered and washed several times with deionized water. It was dried at 50 °C for 24 h. In the final step, 1 mmol (0.187 g) of Cu(NO_3_)_2_ and 200 mg of the ND@Tannicacid were separately dissolved in 10 mL of ethanol and subsequently added together slowly. The resulting mixture was then allowed to react for 6 h at ambient temperature. The produced catalyst was washed several times with ethanol and water and dried at 50 °C for 24 h (Fig. 1).

### General procedure for the synthesis of 1,4-benzodiazepin derivatives

**Figure 2:**
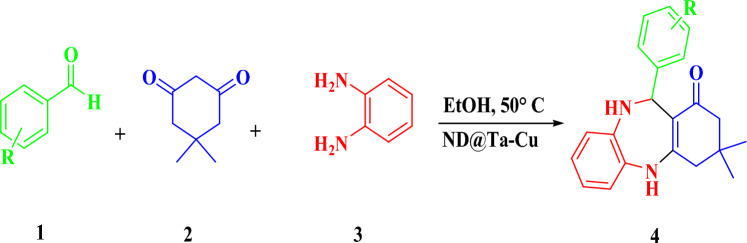
Synthesis process of 1,4-benzodiazepine derivatives in the presence of synthesized catalyst.

In a flask, 1 mmol of ortho-phenylenediamine (0.108 g), 1 mmol of dimedone (0.140 g), 1 mmol of benzaldehyde derivatives, and 10 mg of the ND@Tannicacid-Cu was dissolved in 5 mL of EtOH and stirred at 50 °C (Fig. 2). The completion of the reaction was monitored by thin layer chromatography (TLC). After that, the catalyst was easily separated by filtration. The pure product obtained from the reaction mixture was recrystallized by hot ethanol. All of the products were known compounds, which were identified through the characterization of their melting points as provided in Table 3. This identification was conducted by comparing the melting points of the products with those of authentic literature samples. In certain instances, the identification was further supported by analyzing the^1^H-NMR spectral data of the compounds.

## Results and discussion

### Characterization of the as-prepared ND@Tannicacid-Cu nanocatalyst

#### FT-IR analysis

**Figure 3:**
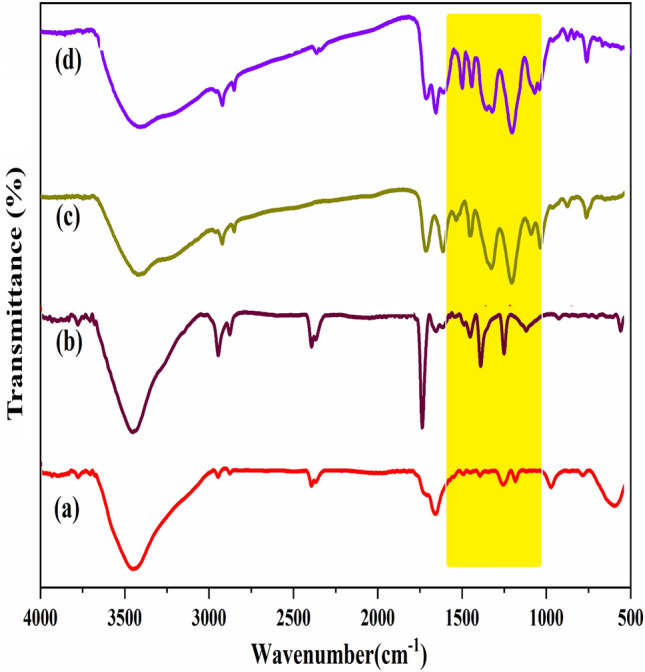
FT-IR spectra of (**a**) ND, (**b**) ND-COOH, (**c**) ND@Tannicacid, and (**d**) ND@Tannicacid-Cu catalyst.

The ND@Tannicacid-Cu catalyst was successfully prepared and characterized by several techniques. The FT-IR spectra of (a) ND, (b) ND-COOH, (c) ND@Tannicacid, and (d) ND@Tannicacid-Cu catalyst are shown in Fig. 3. As depicted in Fig. 3a, the diamond lattice vibration observed at a wavenumber of 1332 cm^−1^ is associated with the first order Raman band and is usually not observed in the FT-IR. Nevertheless, this particular band has been observed in multiple studies, where its presence has been attributed to the disruption of symmetry in the diamond C–C bond near the surface caused by the surface groups. Moreover, the broad absorption band observed within the frequency range of 1720–1780 cm^−1^ can be attributed to the stretching modes of C = O bonds, which are induced by oxidation treatments that are employed during the cleaning process of non-diamond carbon from the NDs. Finally, the observation of C-H bending modes around 1460 cm^−1^ is made in the presence of hydrogenated groups on the surface of the NDs. In Fig. 3b, the absorption spectrum at 1720 cm^−1^ exhibits the vibrational stretching of the carbonyl linkage (C = O)^36^. The bands observed at 2917 cm^−1^ are attributed to the asymmetric and symmetric stretching vibrations of C–H bonds^37^. Furthermore, the strong peak that appears at 3424 cm^−1^ describes the asymmetric stretching vibration of the O–H group. All of these various explanations contribute to the phenomenon of diamond nanoparticle carboxylation. Figure 3c depicts the FT-IR spectrum of the ND@Tannicacid. The strong peak at 1745 cm^−1^ demonstrates the stretching vibration of the carbonyl group of ester^38^. Besides, the absorption band in the region of 3223 cm^−1^ is related to the symmetric and asymmetric stretching vibration of the aromatic C-H bond, which is the reason for the interaction between tannic acid and nanodiamonds (Fig. 3d).

#### Morphological observations

**Figure 4:**
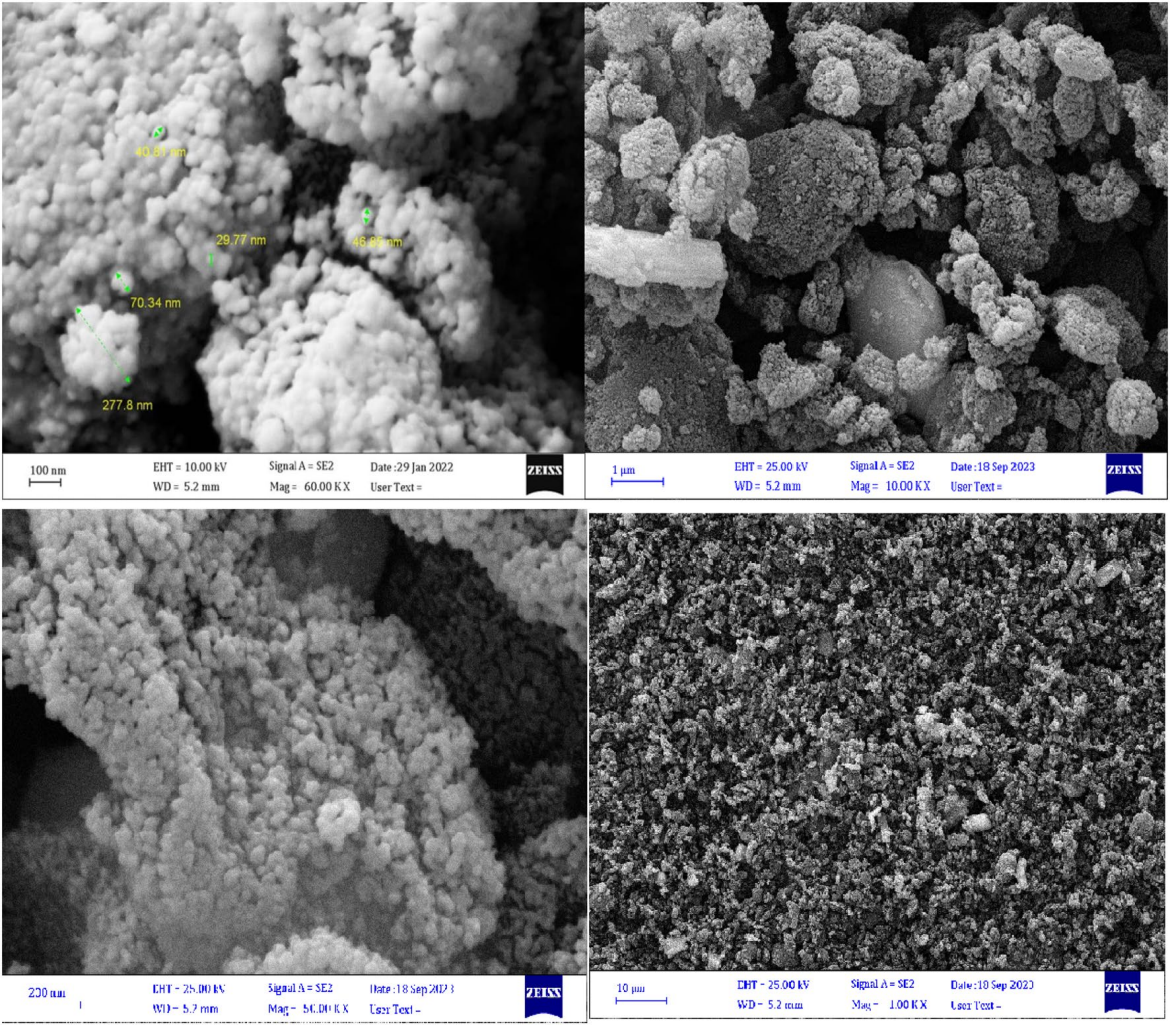
SEM images of the ND@Tannicacid-Cu catalyst with different scale bars.

The morphology and size details of the as-prepared catalyst were investigated by SEM measurements, as illustrated in Fig. 4. The SEM images of the ND@Tannicacid-Cu nanocatalyst demonstrate that the particle shape is spherical and the particle size distribution is uniform with an average size of 35 ± 10 nm.

#### EDX and elemental mapping analyses

**Figure 5:**
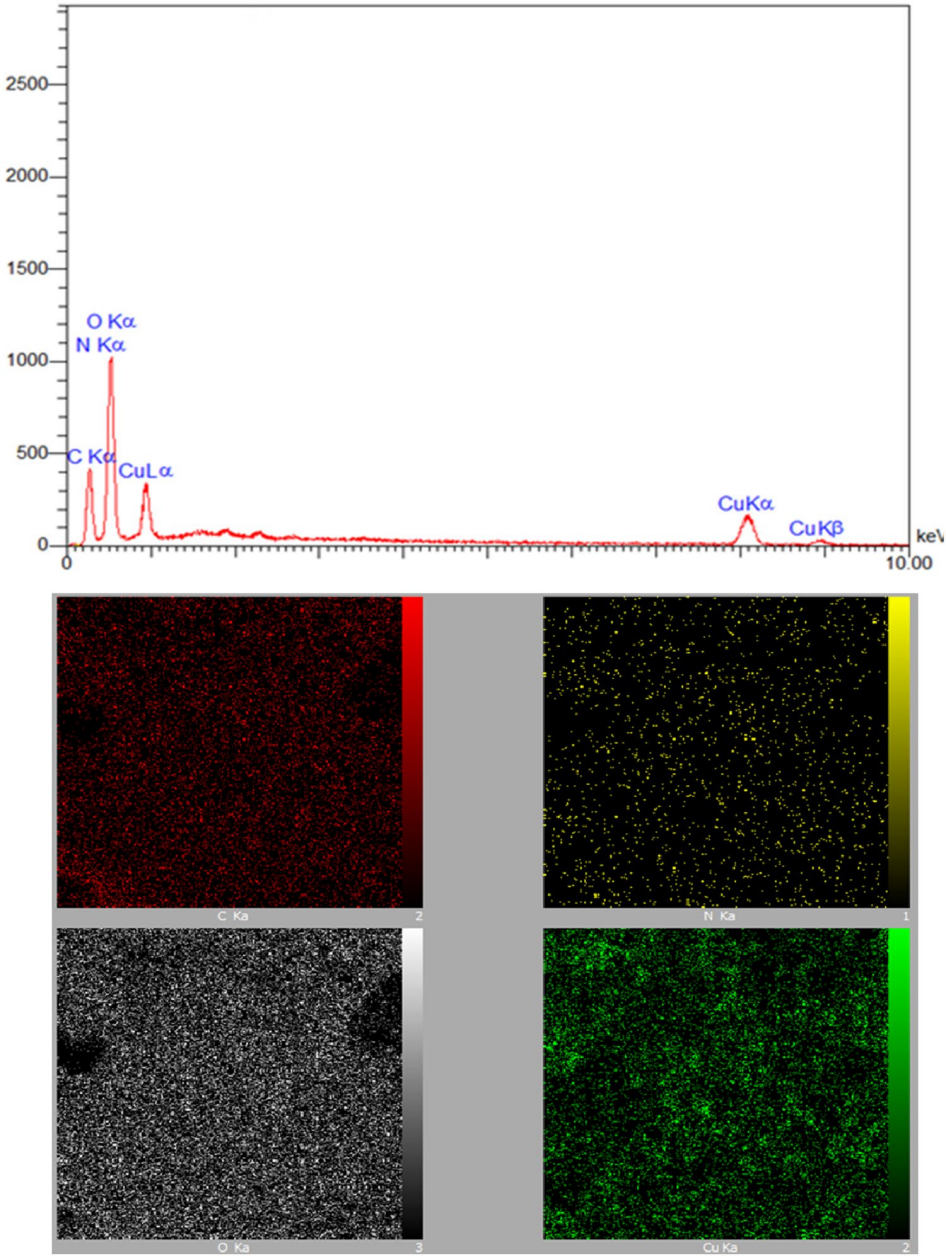
EDX and elemental mapping analyses of ND@Tannicacid-Cu nanocatalyst.

The findings from the analysis of the ND@Tannicacid-Cu nanoparticles, utilizing EDX-mapping, are visually presented in Fig. 5. It confirms the presence of C, O, N, and Cu elements in the structure of the as-prepared catalyst. It can be concluded that the elemental mapping of components in the ND@Tannicacid-Cu catalyst conforms to the percentages reported by the EDX analysis.

#### XRD data

**Figure 6:**
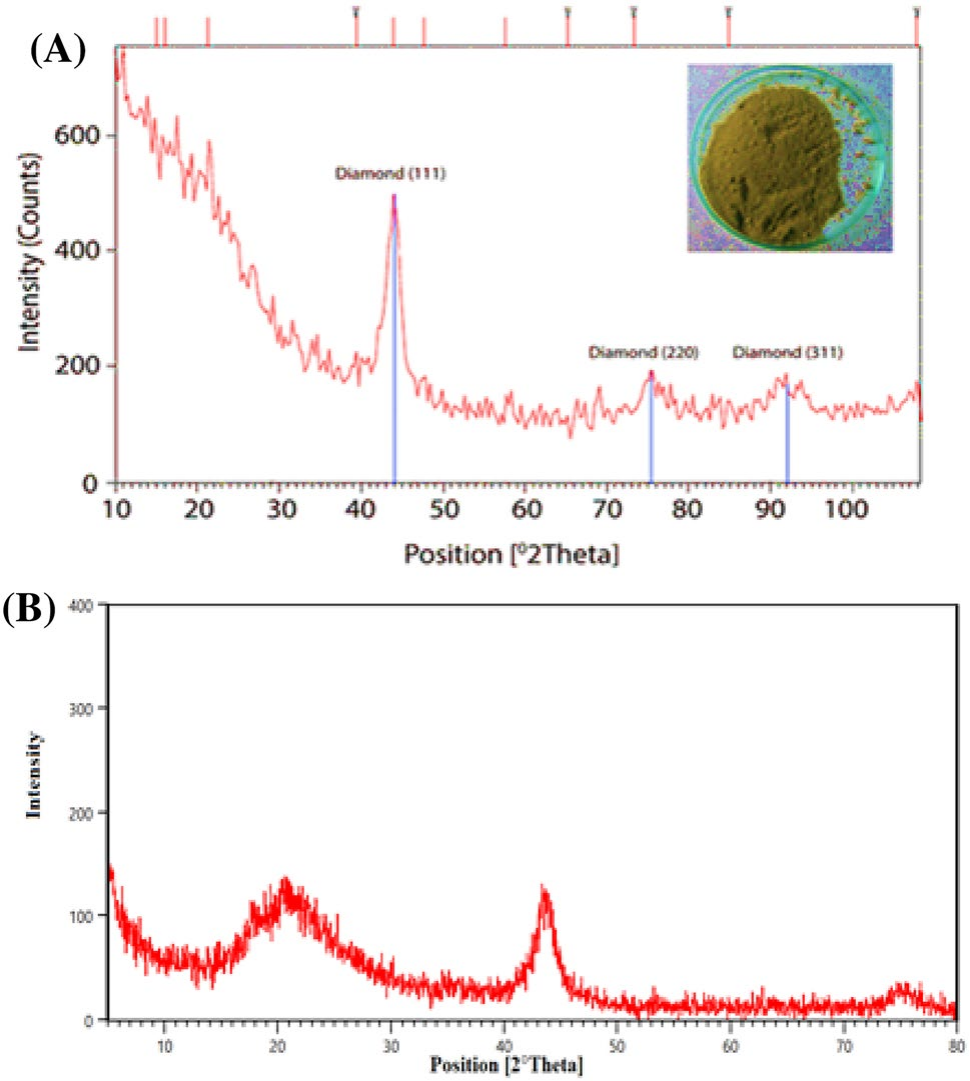
XRD diffraction patterns of (**A**) ND crystals and (**B**) carboxylic modified ND@Tannicacid-Cu.

The XRD technique was scrutinized over the scanning span (5° ≤ 2θ ≤ 80°) in order to validate the crystalline configuration of the catalyst (Fig. 6). The diffraction pattern contains three peaks at 2θ $\sim$ 43.9°, 75.3°, and 91.5° corresponding to X‐ray diffraction on the (111), (220), and (311) planes of diamond crystals (Fig. 6a)^39,40^. According to the Fig. 6b, the carboxylic modified ND@Tannicacid-Cu shows typical peaks at 2θ $\sim$ 22°، 44.2° and 75.8° (2θ), which attributed to the (111) and (220) plane diffractions.

### Catalytic performance of ND@Tannicacid-Cu catalyst

We conducted optimization on the catalytic efficacy of various catalysts, such as ND, ND-OH, ND-COOH, Tannic acid, Cu(NO_3_)_2_, ND@Cu, ND@Tannicacid, and ND@Tannicacid-Cu (Table 1, Entries 1–8). These catalysts were utilized in a one-pot three component reaction involving o-phenylenediamine 1 (1 mmol, 0.108 g), diamond 2 (1 mmol, 0.140 g), and 4-nitrobenzaldehyde 3 (1 mmol) in EtOH at 50 °C as a model reaction (Table 1). Afterward, the effects of temperature on the reaction efficiency were investigated (Table 1, Entries 9–12). When the reaction reached its completion under ambient conditions, an observation of 70% yields was made. As the temperature escalated, there was a corresponding rise in the reaction yields. Subsequently, we conducted an examination on the impacts of different solvents in the reaction (Entries 16–13). Drawing on the data acquired from Table 1, it can be deduced that EtOH solvent is the optimal choice for obtaining the highest yields in the reaction. Finally, we studied the effects of the amount of catalyst on the reaction (Entries 17–20). It has been determined that the utilization of 10 mg of ND@Tannicacid-Cu nanocatalyst is adequate for the purpose of accomplishing the reaction within 10 min, resulting in a yield of 98% in 5 mL of EtOH at reflux temperature. This particular volume of EtOH serves effectively as an environmentally friendly solvent when maintained at the ambient temperature of the surrounding environment.

**Table 1:** Optimizing of the reaction conditions in the synthesis of 1,4-benzodiazepin derivatives.

Entry	Catalyst	Catalyst amount	Solvent	Temp (°C)	Time(min)	Yield (%)
1	ND	10 mg	EtOH	Reflux	12h	45
2	ND-OH	10 mg	EtOH	Reflux	5h	57
3	ND-COOH	10 mg	EtOH	Reflux	3h	61
4	Tannic acid	10 mg	EtOH	Reflux	6h	50
5	Cu (NO_3_)_2_	10 mg	EtOH	Reflux	9h	40
6	ND@Cu	10 mg	EtOH	Reflux	3h	60
7	ND@Ta	10 mg	EtOH	Reflux	3h	50
8	ND@Ta-Cu	10 mg	EtOH	50	10	96
9	ND@Ta-Cu	10 mg	EtOH	r.t	10	70
10	ND@Ta-Cu	10 mg	EtOH	50	10	96
11	ND@Ta-Cu	10 mg	EtOH	60	10	96
12	ND@Ta-Cu	10 mg	EtOH	Reflux	10	98
13	ND@Ta-Cu	10 mg	H_2_O	50	2h	80
14	ND@Ta-Cu	10 mg	DMF	50	10h	70
15	ND@Ta-Cu	10 mg	CH_3_CN	50	6h	70
16	ND@Ta-Cu	10 mg	EtOH	50	10	96
17	ND@Ta-Cu	5 mg	EtOH	50	10	77
18	ND@Ta-Cu	15 mg	EtOH	50	10	90
19	ND@Ta-Cu	20 mg	EtOH	50	10	88
20	ND@Ta-Cu	10 mg	–	50	6h	60

### Comparison of different Ar group effects in the presence of the ND@Tannicacid-Cu

In Table 2, a comparative analysis was conducted between the current study and previous reports pertaining to the synthesis of 4d. The outcomes undeniably showcase the advantageous nature of the current study in terms of time efficiency, energy conservation, and substantial product yields. Furthermore, the potential for nanocatalyst reusability is also evident. With great pleasure, it was noted that a remarkable level of effectiveness, reaching a maximum of 96%, could be attained for 10 min. This resulted in the production of a turnover number (TON) of 160 × 10^3^ and a turnover frequency (TOF) of 941 × 10^3^ (Table 2, Entry 4).

**Table 2:** Comparison of some catalyst’s effects with ND@Tannicacid-Cu on the model reaction.

No	Ar group	Product	Time (min)	Yield^a^ (%)	TON	TOF (h^−1^)	Mp (°C)	
							Observed	Literature
1	Phenyl	4a	12	94	157 × 10^3^	785 × 10^3^	245–246	245–247^43^
2	4-Methylphenyl	4b	15	90	150 × 10^3^	600 × 10^3^	215–217	217–219^44^
3	2-Methoxyphenyl	4c	15	90	150 × 10^3^	600 × 10^3^	217–219	214–217^45^
4	4-Nitrophenyl	4d	10	96	160 × 10^3^	941 × 10^3^	276–279	274–275^42^
5	3-Nitrophenyl	4e	12	93	155 × 10^3^	775 × 10^3^	142–143	144–146^42^
6	4-Bromophenyl	4f.	15	90	150 × 10^3^	600 × 10^3^	292–294	294–296^45^
7	4-Chlorophenyl	4 g	15	88	146 × 10^3^	584 × 10^3^	236–237	235–237^41^
8	2-Chlorophenyl	4 h	12	90	150 × 10^3^	750 × 10^3^	233–234	233–235^32^
9	4-Hydroxyphenyl	4i	20	86	143 × 10^3^	433 × 10^3^	231–234	230–233^32^
10	4-Methoxyphenyl	4j	15	89	148 × 10^3^	592 × 10^3^	230–232	229–231^32^

### Comparative studies

After the optimization of the reaction parameters, an investigation was conducted to ascertain the breadth and universality of these optimized conditions in the synthesis of a diverse range of 1,4-benzodiazepine derivatives. The results presented in Table 3 demonstrate that all the desired products were attained with excellent efficiency following the appropriate duration of the reaction.

**Table 3:** Synthesis of 1,4-benzodiazepine derivatives catalyzed by ND@Tannicacid-Cu catalyst.

Entry	Catalyst	Loading	Solvent	Temp (°C)	Time(min)	Yield	Ref
1	NiO-SiO_2_ NCs	50 mg	EtOH	MW,80	10	98	^45^
2	Fe_3_O_4_@chitosan	30 mg	EtOH	r.t	70	94	^46^
3	CoFe_2_O_4_@GO-K22-Ni	30 mg	H_2_O	60	10	95	^32^
4	Gr@TiO_2_ NCs	140 mg	EtOH	r.t	100	75	^47^
5	Oxalic acid	40 mol%	H_2_O	100	120	94	^48^
6	ND-COOH	10 mg	EtOH	50	10	70	This work
7	ND@Tannicacid	10 mg	EtOH	50	10	90	This work
8	ND@Ta-Cu	10 mg	EtOH	50	10	96	This work

### Suggested reaction mechanism

**Figure 7:**
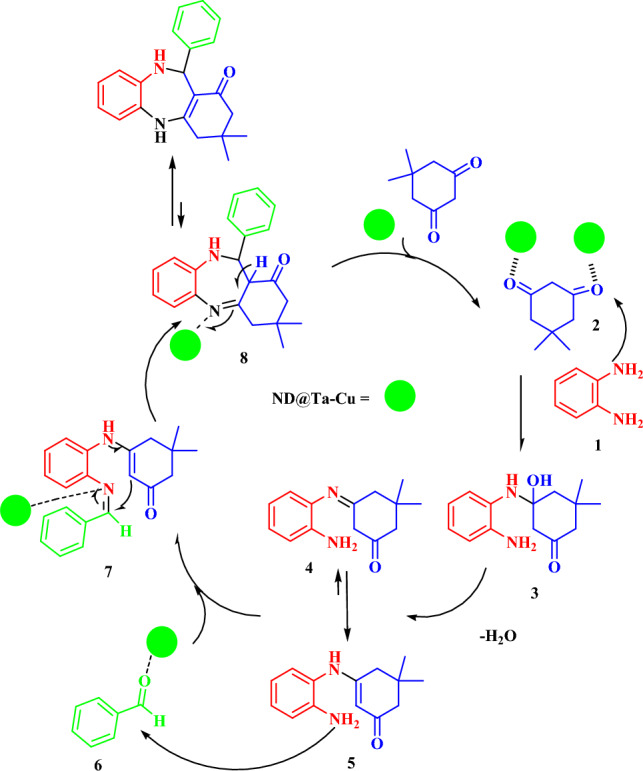
The possible reaction mechanism for the synthesis of 1,4-benzodiazepine derivation catalyzed by ND@Tannicacid-Cu nanocatalyst.

Based on the results of recent studies, we presented a proposed mechanism for the synthesis of benzodiazepines using ND@Tannicacid-Cu catalyst (Fig. 7)**,** the interaction between the oxygen atom of dimedone and the active sites on the surface of the catalyst occurs through the utilization of lone pairs of electrons. Additionally, the carbonyl group of dimedone is attacked by the NH_2_ group of ortho-phenylenediamine, resulting in the elimination of H_2_O and the formation of the intermediate imine 4. Subsequently, 1,3-hydrogen shift takes place, leading to the creation of the tautomeric enamine 5. The NH_2_ group of the enamine intermediate 5 would subsequently engage in a reaction with the activated carbonyl group of the aromatic aldehyde 6 in order to produce the corresponding imine 7. The imine 7 would then experience an intramolecular cyclization, resulting in the formation of the desired benzodiazepine ring with a seven-membered structure.

### Recyclability of ND@Tannicacid-Cu nanocatalyst

**Figure 8:**
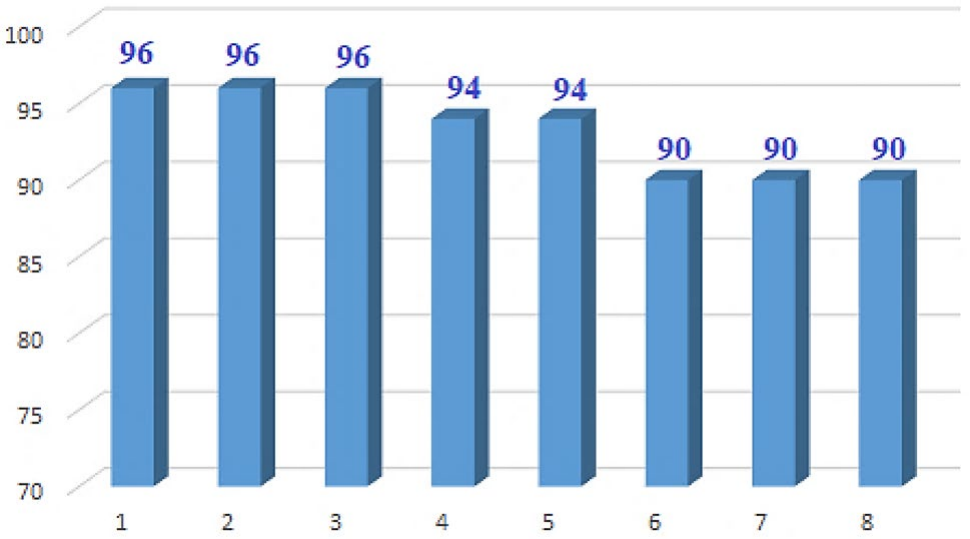
Reusability of the ND@Tannicacid-Cu nanocatalyst.

The reusability of the catalyst is one of the main advantages, allowing it to be used in commercial applications. Thereafter, the reusability of ND@Tannicacid-Cu nanocatalyst was investigated in model reactions (Fig. 8). After the completion of the reaction, the nanocatalyst was filtered and washed several times with diethyl ether, dried and reused in subsequent reactions. It was observed that the catalyst could be reused at least 8 times without significant loss in product yields.

## Conclusions

In summary, functionalization of diamond nanoparticles with copper tannic acid has been synthesized by esterification process and completely characterized by several analysis. Afterwards, the catalytic activity of the ND@Tannicacid-Cu in the synthesis of 1,4 benzodiazepine derivatives was investigated. The products were obtained in excellent yield under mild reaction conditions and compatible with green chemistry. The as-prepared nanocatalyst was easily filtered and used for eight times without significant decrease in its catalytic activity. This is the first report on the design, synthesis, and characterization of the present nanocatalyst in synthesis of 1,4-benzodiazepines derivatives.

## Supplementary Information

Supplementary Information.

## Data Availability

The datasets used and/or analyzed during the current study available from the corresponding author on reasonable request.
