# Supplementary material for: Preparation of nanodiamond anchored on copper tannic acid as a heterogenous catalyst for synthesis of 1,4-benzodiazepines derivatives
Source: Sci Rep. 2024 Apr 15;14:8655. doi: 10.1038/s41598-024-58563-0 (PMC11018864; doi:10.1038/s41598-024-58563-0)
Supplement: Supplementary file 1 — Supplementary Information. [file 41598_2024_58563_MOESM1_ESM.docx]

**Supporting Information**

**Preparation of nanodiamond anchored on copper tannic acid as a heterogenous catalyst for synthesis of 1,4-benzodiazepines derivatives**

**Reza Ghalavand^a^, Hossein Ghafuri ^a,*^, and Hadi Hassani Ardeshiri^a^**

*^a^Catalysts and Organic Synthesis Research Laboratory, Department of Chemistry, Iran University of Science and Technology, Tehran16846-13114, Iran*

*^*^Catalysts and Organic Synthesis Research Laboratory, Department of Chemistry, Iran University of Science and Technology, Tehran16846-13114, Iran.*

| **Page** | **Table of Content** |
| --- | --- |
| P1 | Title page |
| P2 | Data for 11-(4-methylphenyl)-2,3,4,5,10,11-hexahydro-1H-dibenzo [b,e] [1,4] diazepin-1-one (4b) |
| P3 | Figure S2. Mini size ^1^H NMR spectrum of (4b) compound. |
| P4 | Spectroscopic data for 11-(4-methoxylphenyl)-2,3,4,5,10,11-hexahydro-1H-dibenzo [b,e] [1,4] diazepin-1-one (4J) |
| P5 | Figure S4. Mini size ^1^H-NMR spectrum of (4j) compound. |
| P6 | Figure S5. 1H-NMR spectrum of 11-(4-chlorophenyl)-2,3,4,5,10,11-hexahydro-1H-dibenzo [b,e] [1,4] diazepin-1-one (4J). |

*Spectroscopic data for 11-(4-methylphenyl)-2,3,4,5,10,11-hexahydro-1H-dibenzo [b,e] [1,4] diazepin-1-one (4b)*

Yellow Solid, M.p. 215-217 °C; IR (KBr, υ, cm^-1^): 3382, 3321, 3207, 2962, 1677, 1660  ^1^H NMR (500 MHz, CDCl_3_): δ 1.062 (s, 3H, CH_3_), 1.142 (s, 3H, CH_3_), 2.182 (s, 3H, CH_3_), 2.201-2.329 (d, 3H, -CH_2_-), 2.452-2.613 (d, 2H, -CH_2_-), 5.958 (s, 1H, CH-), 6.430 (s, 1H, -NH-), 6.766 (m, 4H, ArH), 7.144-7.163 (m, 2H, ArH), 7.376-7.396 (m, 2H, ArH), 8.697 (s, 1H, -NH-), ^13^C NMR (125 MHz, CDCl_3_): δ 20.944, 24.784, 27.752, 28.786, 30.890, 32.235, 45.816, 49.752, 57.575, 110.460, 120.316, 121.285, 121.489, 123.781, 125.733, 129.583, 130.453, 146.442, 154.295, 193.782 (Fig. 7).
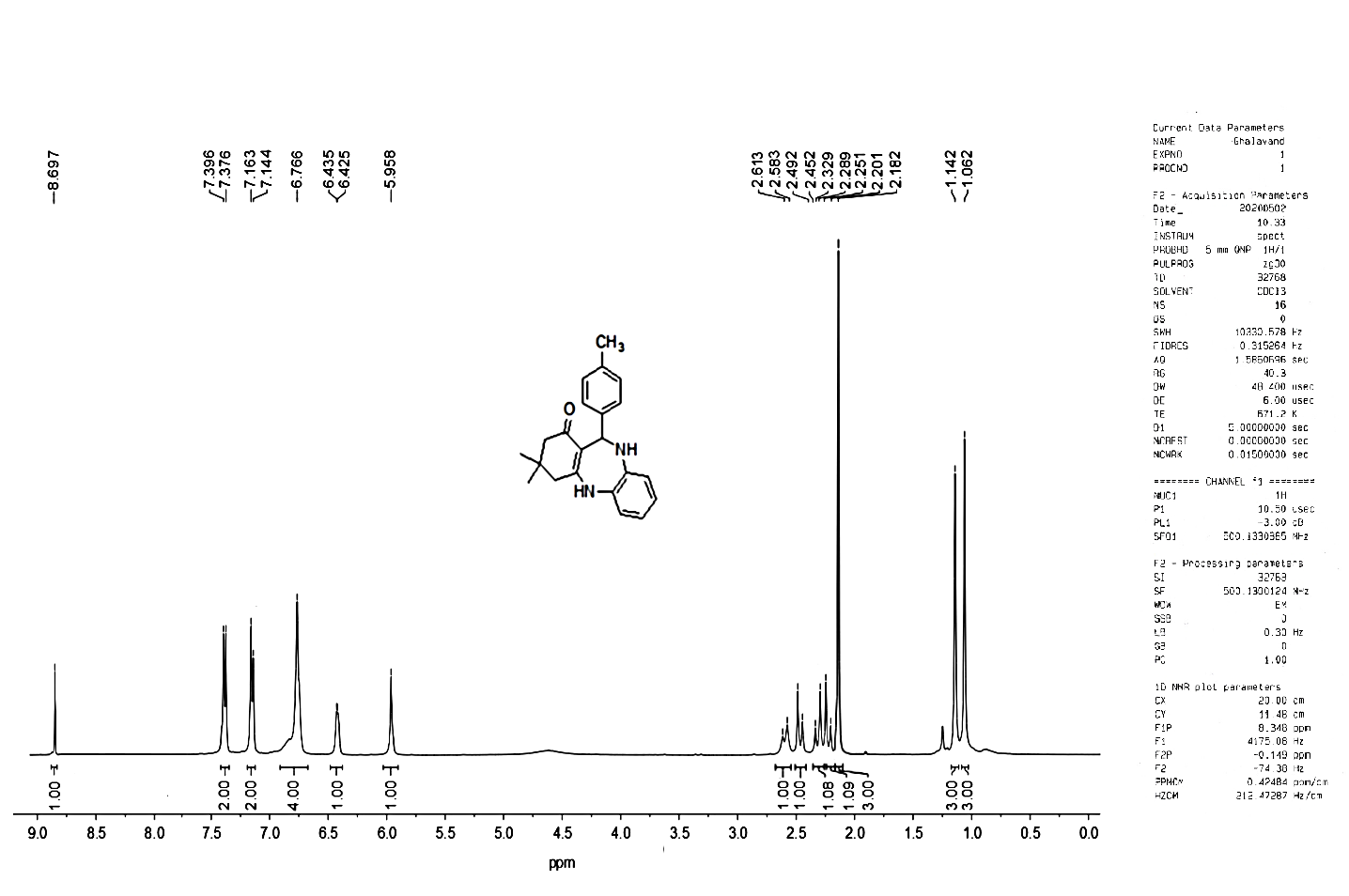


**Figure S1.** ^1^H-NMR spectrum of (**4b**) compound.


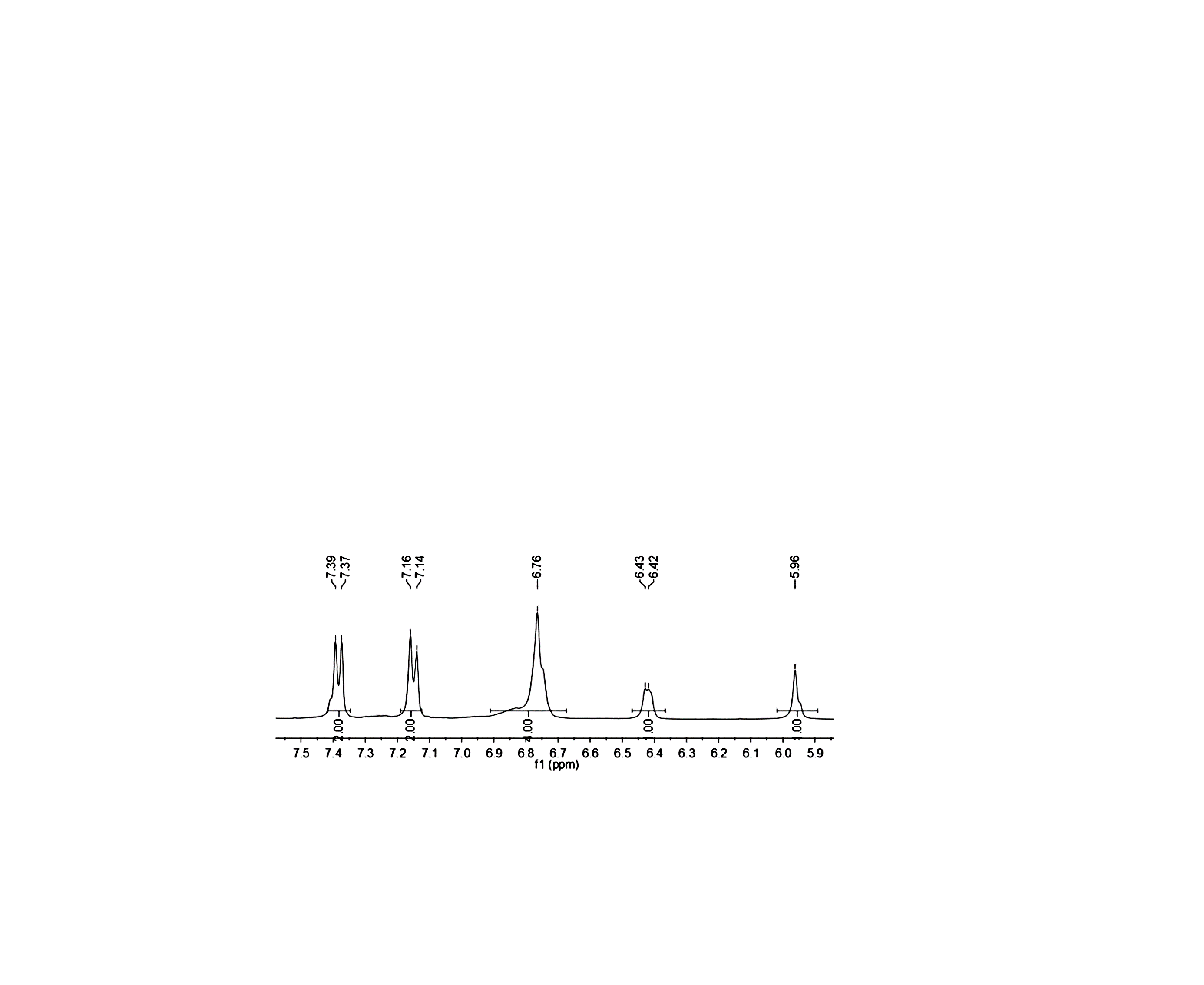


**Figure S2.** Mini size ^1^H NMR spectrum of (**4b**) compound.

*Spectroscopic data for 11-(4-methoxylphenyl)-2,3,4,5,10,11-hexahydro-1H-dibenzo [b,e] [1,4] diazepin-1-one (4J)*

Yellow Solid, M.p. 230-232 °C; IR (KBr, υ, cm-1): 3396, 3330, 3220, 2966, 1687, 1654 1H NMR (500 MHz, CDCl3): δ 1.052 (s, 3H, CH3), 1.111 (s, 3H, CH3), 2.201-2.265 (d, 3H, -CH2-), 2.283-2.258 (d, 2H, -CH2-), 3.662 (s, 3H, -OCH3), 5.905-5.964 (d, 1H, CH-), 6.443-6.464 (m, 1H, -NH-), 6.620 (m, 2H, ArH), 6.676 (m, 3H, ArH), 6.972-6.992 (m, 3H, ArH), 7.376-7.396 (m, 2H, ArH), 8.697 (s, 1H, -NH-) (Fig. 8).


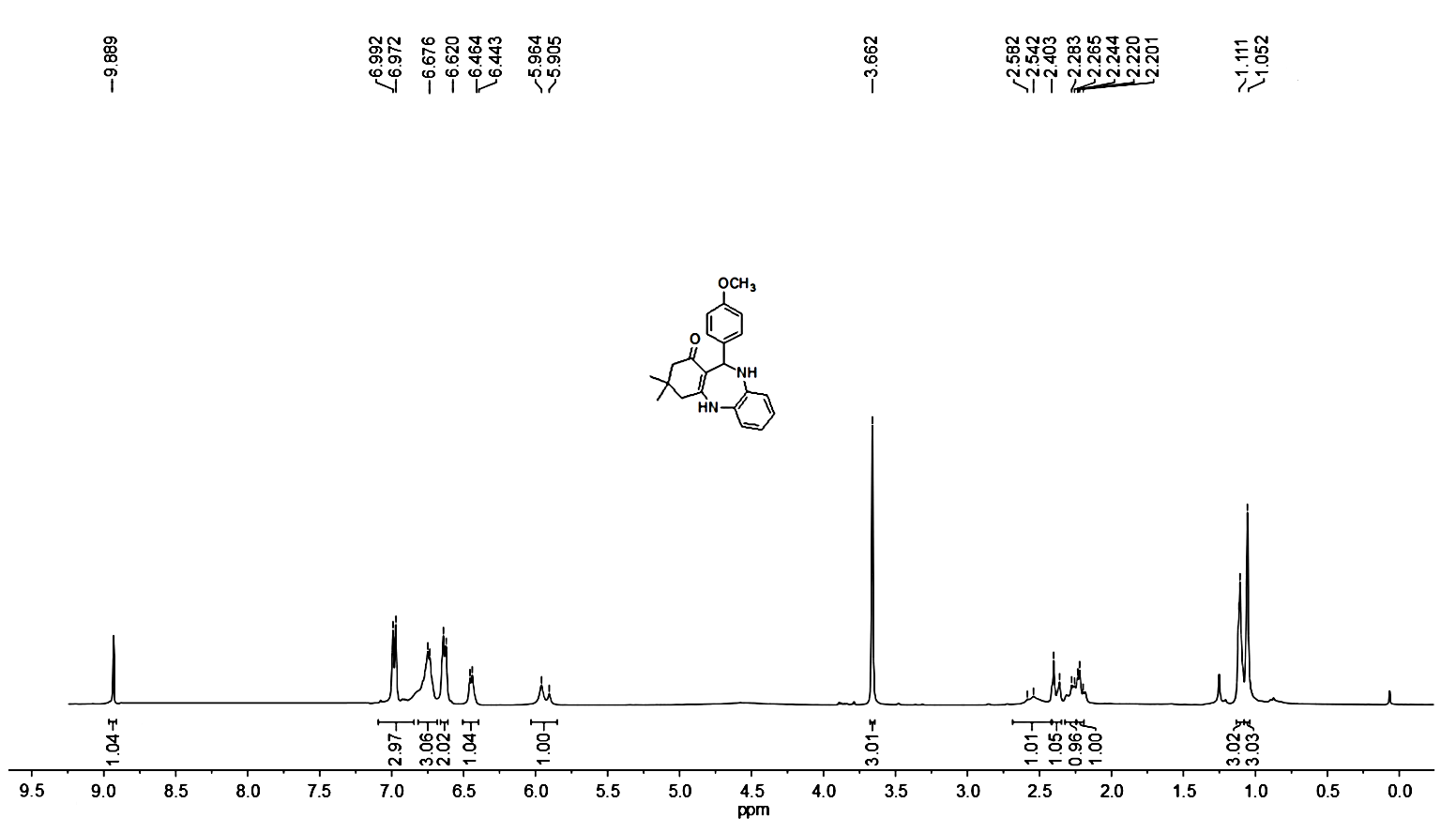


**Figure S3.** ^1^H-NMR spectrum of (**4j**) compound .


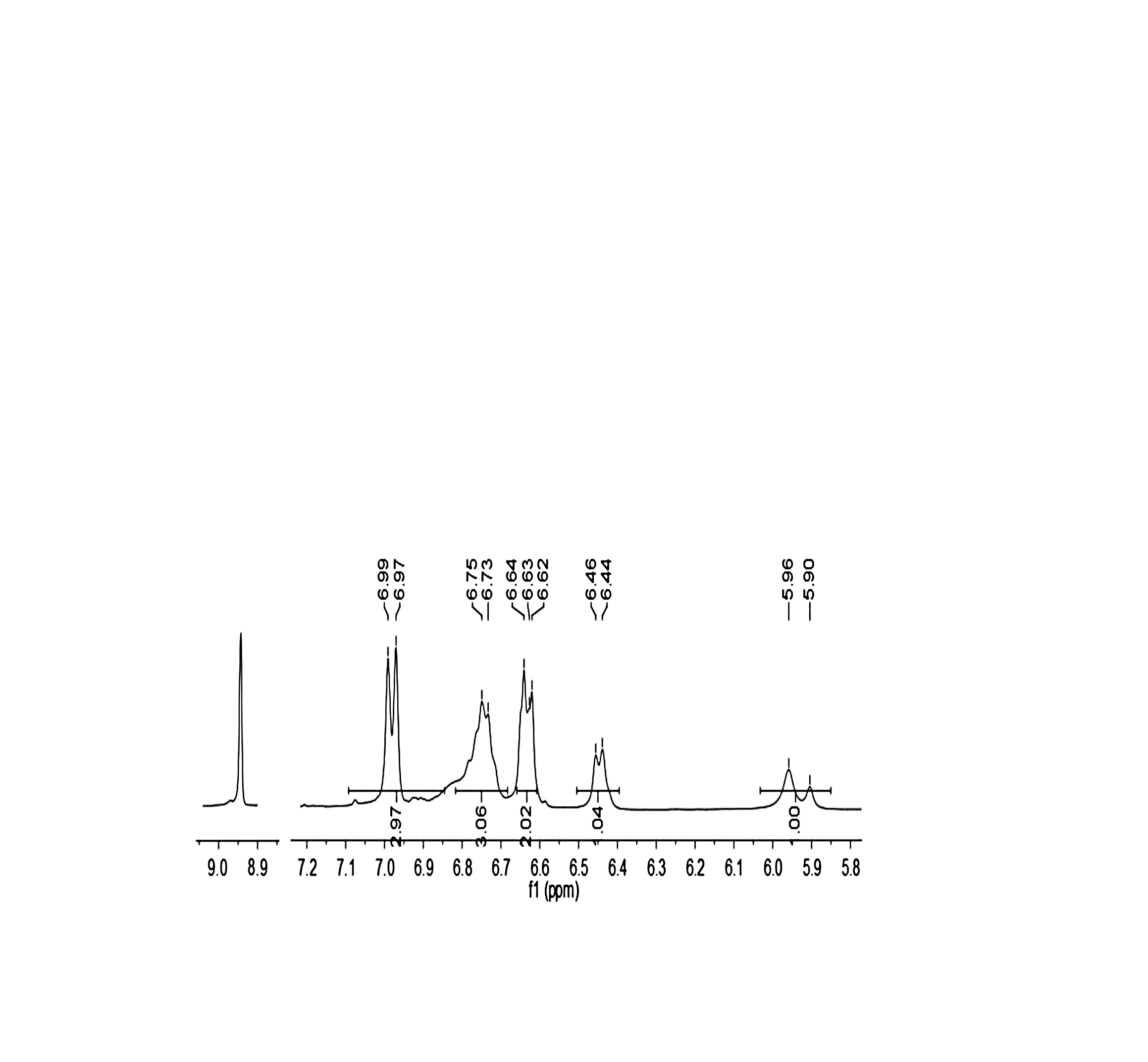


**Figure S4.** Mini size ^1^H-NMR spectrum of (**4j**) compound.


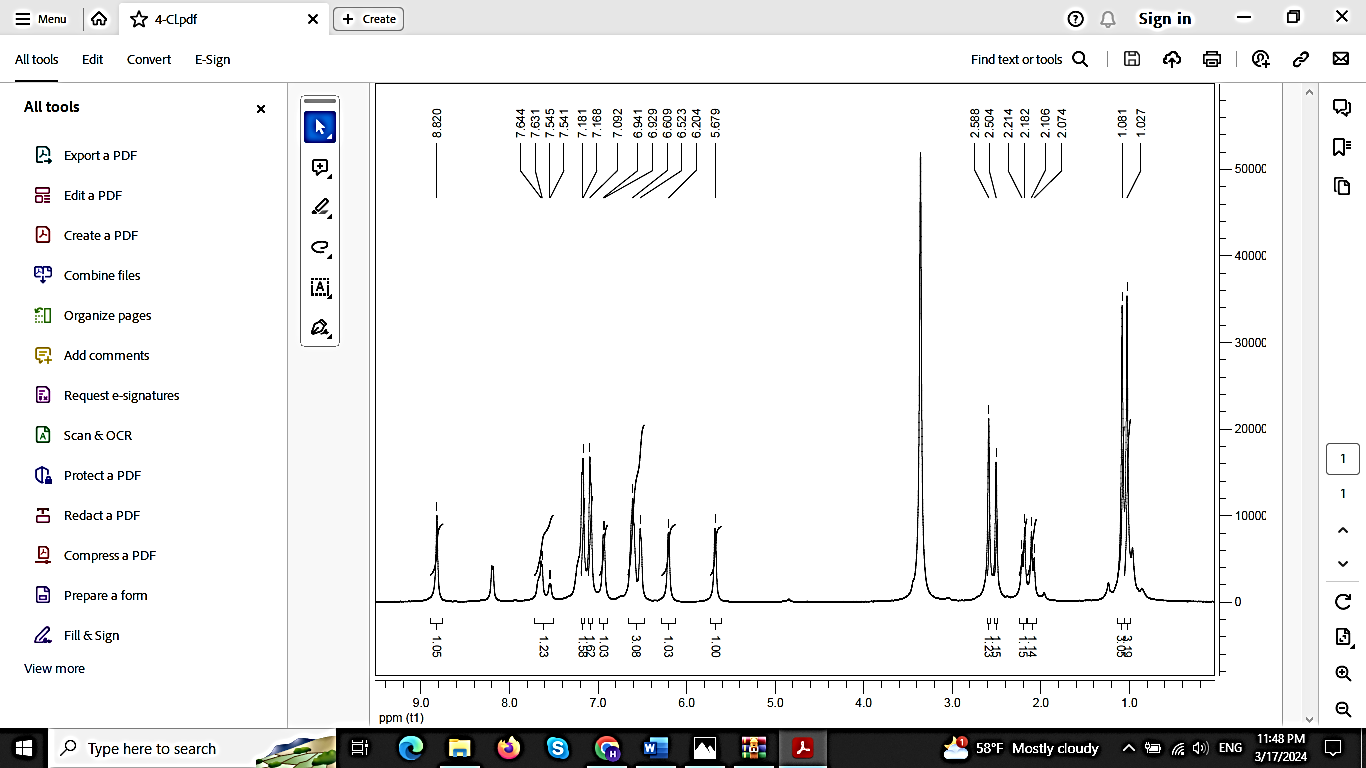


**Figure S5.** ^1^H-NMR spectrum of 11-(4-chlorophenyl)-2,3,4,5,10,11-hexahydro-1H-dibenzo [b,e] [1,4] diazepin-1-one (4J).
